# Evaluation of the Nasopharyngeal Microbiota in Beef Cattle Transported to a Feedlot, With a Focus on Lactic Acid-Producing Bacteria

**DOI:** 10.3389/fmicb.2019.01988

**Published:** 2019-09-06

**Authors:** Samat Amat, Devin B. Holman, Edouard Timsit, Timothy Schwinghamer, Trevor W. Alexander

**Affiliations:** ^1^Agriculture and Agri-Food Canada, Lethbridge Research and Development Centre, Lethbridge, AB, Canada; ^2^Department of Production Animal Health, Faculty of Veterinary Medicine, University of Calgary, Calgary, AB, Canada; ^3^Agriculture and Agri-Food Canada, Lacombe Research and Development Centre, Lacombe, AB, Canada; ^4^Simpson Ranch Chair in Beef Cattle Health and Wellness, University of Calgary, Calgary, AB, Canada

**Keywords:** bovine respiratory disease, nasopharyngeal microbiota, auction market, feedlot cattle, respiratory pathogens, lactic acid-producing bacteria, antimicrobial activity

## Abstract

The nasopharyngeal (NP) microbiota is important in defining respiratory health in feedlot cattle, with certain NP commensals potentially protecting against bovine respiratory disease (BRD) pathogens. In the present study, we evaluated longitudinal changes in the NP microbiota with a focus on lactic acid-producing bacteria (LAB) and their linkage with BRD-associated bacteria in steers (*n* = 13) that were first transported to an auction market, and then to a feedlot. Deep nasopharyngeal swabs were collected at the farm before transportation to the auction market (day 0), at feedlot placement (day 2), and 5 (day 7) and 12 (day 14) days after feedlot placement. Swabs were processed for the assessment of the NP microbiota using 16S rRNA gene sequencing, and for the detection of *Mannheimia haemolytica, Pasteurella multocida*, and *Histophilus somni* by culturing. Possible associations among the top 15 most relatively abundant bacterial genera were predicted using a stepwise-selected generalized linear mixed model. Correlations between LAB and BRD-associated *Pasteurellaceae* families were also assessed. In addition, antimicrobial activity of selected LAB isolates against *M. haemolytica* was evaluated *in vitro*. A noticeable shift was observed in the NP microbial community structure, and in the relative abundance of LAB families as a result of auction market exposure, transport and feedlot placement. Varying degrees of positive or negative associations between the 15 most relatively abundant genera were observed. Many of the LAB families were inversely correlated with the BRD-associated *Pasteurellaceae* family as the cattle were transported to the auction market and then to the feedlot. Nearly all steers were culture-negative for *M. haemolytica* and *H. somni*, and *P. multocida* became less prevalent after feedlot placement. Isolates from the *Lactobacillaceae, Streptococcaceae*, and *Enterococcaceae* families inhibited the growth of *M. haemolytica*. The results of this study indicated that the NP microbiota became more diverse with an increase in microbial richness following transport to an auction market and feedlot. This study provides evidence of potential cooperation and exclusion taking place in the respiratory microbial community of cattle which may be useful for developing microbial-based strategies to mitigate BRD.

## Introduction

Bovine respiratory disease (BRD) is the most common endemic disease in feedlot cattle, accounting for ~$4 billion in annual losses to the United States feedlot industry due to the costs of treatment, prevention, and lost productivity (Cernicchiaro et al., 2013; Johnson and Pendell, 2017). Although BRD is a multifactorial disease with a multitude of stressors that predispose cattle to viral and bacterial infections, bacterial pathogens are the principal agent in the pathogenesis of BRD. The major bacterial species involved in BRD are *Mannheimia haemolytica, Pasteurella multocida, Histophilus somni*, and *Mycoplasma bovis*. These are opportunistic pathogens that are often present in the nasopharynx of the healthy cattle as part of the commensal microbiota (Griffin et al., 2010). However, when the host experiences compromised immunity due to viral infection, or stressors such as maternal separation, transportation, and commingling at auction markets and after feedlot placement, these opportunistic bacteria can proliferate in the nasopharynx and translocate into the lung where they cause bacterial pneumonia (Rice et al., 2007).

Bacterial pneumonia most often occurs within the first week of feedlot placement (Babcock et al., 2010). Due to the segmented nature of beef production and large differences in the operational scale of cow-calf producers and commercial feedlots in North America, beef calves are frequently transported to a feedlot for finishing, either directly from a farm, or first through delivery to an auction market. Some studies have shown a negative impact of shipment and commingling at an auction market on the incidence of BRD (Step et al., 2008). The effects of these risk factors for BRD vary based on the season of the shipment (Hay et al., 2016) and distance of transportation between farm to the feedlot (Cernicchiaro et al., 2012; Hay et al., 2014). Currently, antibiotics are the most common management practice used to prevent (metaphylaxis) BRD in feedlot cattle. Although usually effective, the large-scale use of antibiotics has come under greater scrutiny due to an increase in antibiotic-resistant BRD pathogens (Snyder et al., 2017; Timsit et al., 2017). Therefore, there is a need for the discovery and development of novel antibiotic alternatives that are effective against BRD pathogens.

Culture-independent approaches have enhanced our understanding of the potential role of nasopharyngeal (NP) microbiota in the respiratory health of cattle (Timsit et al., 2016a). The bovine nasopharynx harbors a relatively rich and diverse microbial community which is dynamic and changes in response to various factors (Holman et al., 2015a, 2017; Timsit et al., 2016b). Previous studies have suggested a possible association between the NP microbiota and development of BRD in feedlot cattle (Holman et al., 2015b; Zeineldin et al., 2017). A disruption in the NP microbiota may result in the loss of resistance to colonization by BRD pathogens or proliferation of existing opportunistic pathogens in the nasopharynx. Thus, maintaining a stable microbial community in nasopharynx of cattle at feedlot placement, and beyond, may decrease the risk of infection by BRD-associated pathogens. Recent studies comparing the respiratory tract microbiota of healthy and BRD-affected feedlot cattle, suggest that certain lactic acid producing bacteria (LAB) present in the nasopharynx and the lung may be important for respiratory health (Holman et al., 2015a; Timsit et al., 2018). This was further confirmed by recent *in vitro* and *in vivo* studies that demonstrated the inhibitory effects of bovine NP-derived *Lactobacillus* spp. against *M. haemolytica* (Amat et al., 2016, 2018). To date, however, LAB abundance within the NP microbiota of cattle has been poorly characterized. More detailed information on these bacteria and the role they may have in respiratory health is important to better understand BRD and to propose alternatives to antibiotic metaphylaxis.

In the present study, we used 16S rRNA gene sequencing to characterize the NP microbiota in beef cattle that were transported from a closed herd to a local auction market where they were held for 48 h, and then transported to a feedlot. The prevalence of bovine respiratory pathogens, including *M. haemolytica, P. multocida*, and *H. somni* was also evaluated during the course of the study using culture-dependent methods. Finally, antimicrobial activity of selected LAB isolates from the nasopharynx of healthy feedlot cattle was tested against *M. haemolytica in vitro*. We hypothesized that transport to and commingling with other cattle at the auction market would increase the prevalence of BRD-associated bacteria and alter the NP microbiota.

## Materials and Methods

### Animal Husbandry and Experimental Design

Thirteen Angus × Herford cross steers (initial body weight 325 ± 54 kg SD) were sourced from a closed herd in Lethbridge, Alberta, Canada, as previously described (Holman et al., 2017). All steers used in the present study were castrated and had no history of antibiotic or hormone treatment, or vaccination. Calves were weaned 41 days prior to shipment to the auction market and were bunk-fed an alfalfa-barley silage mixed diet during this period. On day 0 of the study, calves were transported from the farm to a local commercial auction market using a cattle-hauling trailer (distance of 25 km). At the auction market, the calves were commingled with other cattle and held for 48 h before transport to the feedlot (distance of 8 km) according to regular management operations by the auction market. Approximately 4,000 head of cattle were sold weekly at this auction market during the course of this study. At the feedlot, calves were held in a pen separate from other cattle and fed a typical backgrounding diet which consisted of alfalfa and barley.

### Nasopharyngeal Swab Sampling and Isolation of BRD-Associated Pathogens

Deep NP swabs were collected from all calves on day 0 (at the closed herd farm prior to shipment), 2 (day of feedlot placement), 7, and 14 (i.e., 5 and 12 days after feedlot placement, respectively), as described previously (Holman et al., 2017). Swabs were kept on ice and transported to the lab where they were processed within 1 h of collection. The procedures associated with swab processing for isolation and PCR confirmation of the respiratory pathogens *M. haemolytica, P. multocida, and H. somni* were identical to those described in our earlier study (Holman et al., 2017). Briefly, for each animal at each time point, up to three isolates displaying typical morphology of *M. haemolytica, P. multocida*, and *H. somni* were subcultured and tested by PCR for species confirmation. An animal was considered positive for the respective bacteria if one of the isolates was PCR-positive, and negative if none of the isolates were PCR-positive. Upon plating of the swab suspension consisting of brain heart infusion (BHI) and glycerol (80:20), the suspension, including swabs, were cryopreserved at −80°C for DNA extraction as outlined in Holman et al. (2017).

### 16S rRNA Gene Sequencing and Analysis

The V4 region of the 16S rRNA gene was amplified and sequenced on a MiSeq instrument (Illumina, San Diego, CA, USA) with the MiSeq Reagent Kit v2 as detailed in Holman et al. (2017). The 16S rRNA gene sequences were processed using DADA2 v. 1.10 (Callahan et al., 2016) in R. 3.5.1. Briefly, the forward reads were truncated at 210 bp and the reverse reads at 200 bp. The reads were merged, chimeric sequences removed, and taxonomy assigned to each merged sequence, referred to here as an operational taxonomic units (OTUs) at 100% similarity, using the naïve Bayesian RDP classifier (Wang et al., 2007) and the SILVA SSU database release 132 (Quast et al., 2012). The number of OTUs per sample and the Shannon diversity index were calculated in R using Phyloseq 1.26.0 (McMurdie and Holmes, 2013) and vegan 2.5-3 (Oksanen et al., 2013) was used to determine the Bray-Curtis dissimilarities and Jaccard distances. Samples were randomly subsampled to 14,500 sequences prior to the calculation of Bray-Curtis dissimilarities, Jaccard distances and alpha-diversity measures. Sequences submitted to the NCBI sequence read archive under BioProject accession PRJNA296393; BioSamples SAMN04102441-SAMN04102444; SAMN04102453-SAMN04102464; SAMN04102473-SAMN04102492; SAMN04102524-SAMN04102531; SAMN04102540-SAMN04102547.

### *In vitro* Growth Inhibition of *M. haemolytica* by LAB Isolates

Eight LAB isolates from four genera were evaluated for inhibition of the BRD pathogen *M. haemolytica*. *M. haemolytica* was used as a model BRD pathogen due to its importance in the development of this disease in feedlot cattle. The LAB strains were previously isolated from healthy cattle (Timsit et al., 2016b) and isolates were identified as described by Holman et al. (2015b). The *M. haemolytica* L024A strain was isolated from a BRD-affected steer originating from a commercial feedlot in Alberta, Canada, and was identified as serotype 1 (Klima et al., 2014). Inhibition of *M. haemolytica* L024A was evaluated using an agar slab method as per Dec et al. (2014) with some modifications. Briefly, 100 μL of a 18 h culture of each LAB isolate was grown in Difco Lactobacilli De Man, Rogosa and Sharpe (MRS) broth (BD, Mississauga, ON, Canada), inoculated onto *Lactobacillus* MRS agar and incubated at 37°C with 5% CO_2_ for 24 h. Agar slabs (10 mm in diameter) were cut from the MRS agar after incubation using a sterile hollow punch (Tekton−12 PC Hollow Punch Set−6588) and placed on a lawn of *M. haemolytica* on tryptic soy agar with 5% sheep blood (Dalynn Biologicals) followed by incubation at 37°C with 5% CO_2_ for 24 h. The lawn of *M. haemolytica* was prepared by spread plating of a 100 μL aliquot of *M. haemolytica* culture suspended in Dulbecco's phosphate-buffered saline (pH 7.4) to obtain the target bacterial concentration of 1 × 10^8^ CFU mL^−1^. After 24 h of incubation, the plates were checked for inhibition zones and the results recorded as the mean diameter (mm) of the inhibition zone for three independent experiments.

### Statistical Analysis

The number of OTUs and Shannon diversity index were analyzed by sampling day using a linear mixed model implemented in R with lme4 v 1.1.15 (Bates et al., 2014) with time as the fixed effect and animal as the random effect. Permutational multivariate analysis of variance (PERMANOVA; adonis function; 10,000 permutations) of the Bray-Curtis dissimilarities and Jaccard distances was performed to assess the effect of sampling day on the NP microbial community structure. A pairwise adonis function (Arbizu, 2017) was used to determine which sampling days were most dissimilar from each other and *p*-values corrected for multiple comparisons using Holm's method. Differentially abundant OTUs between sampling times were identified using DESeq2 in R (Love et al., 2014) and corrected for multiple comparisons using the false discovery rate. For this analysis samples were not randomly subsampled but OTUs found in <25% of the samples were removed. The relative abundance of phyla, the *Lactobacillales* order, families within the *Lactobacillales* order, and the 15 most relatively abundant genera were analyzed using generalized liner mixed model estimation procedure (PROC GLIMMIX) in SAS (ver. 9.4, SAS Institute Inc. Cary, NC). The means were compared using LSMEANS statement and significance was declared at *P* < 0·05. Lactic acid-producing bacteria are found in the *Lactobacillales* order, encompassing the following six families: *Aerococcaceae, Carnobacteriaceae, Enterococcaceae, Lactobacillaceae, Leuconostocaceae, and Streptococcaceae* (Mattarelli et al., 2014). Spearman's rank-based correlations between LAB families and the BRD-associated family of *Pasteurellaceae* were calculated using the CORR procedure in SAS with the SPEARMAN option. The *Pasteurellaceae* family was comprised of *Mannheimia* and *Pasteurella* genera. Possible associations among the 15 most relatively abundant genera were predicted using a stepwise-selected GLIMMIX model with beta-binomial distribution. The model used was: logit (Ŷ) = ln (π/(1 – π)) = *b*_0_ + *b*_time_ + *b*_time2_ + *b*_1_ (X_1_) + … + *b*_*n*_ (X_*n*_), where π represents the relative abundance of a bacterial genus (0–1), X_*n*_ represents relative abundance (0–100%) of a bacterial genus *n*. The stepwise selection method involved backward elimination and forward selection to eliminate any variables in the model that have no significant effect (*P* > 0.05) on the predicted outcome.

## Results

### Isolation and Detection of BRD-Associated Pathogens

All cattle remained healthy throughout the study and were not treated for any diseases. We assessed the NP swabs for the presence of *M*. *haemolytica, P*. *multocida*, and *H*. *somni* via culturing (Table 1). Among these three BRD-associated pathogens, *P. multocida* was most frequently isolated, with 6, 2, 4, and 2 animals positive for *P*. *multocida* on days 0, 2, 7, and 14, respectively. None of the experimental animals were positive for *P. multocida* at all four sampling times. *M. haemolytica* was isolated from only a single animal on day 14 and *H. somni* was not detected among any of the cattle at any sampling time.

**Table 1:** Calves (*n* = 13) positive for BRD-associate bacterial pathogens by culturing of nasopharyngeal swabs^a^.

**Sampling time (day)**	***H. somni***	***P. multocida***	***M. haemolytica***
0	–	1, 11, 15, 22, 27, 53	–
2	–	11, 15	–
7	–	11, 13, 23, 27	–
14	–	27, 44	34

a *Numbers represent unique animal identifier*.

### Effect of Transport to the Auction Market and Feedlot on the Structure of the Nasopharyngeal Microbiota

**Figure 1:**
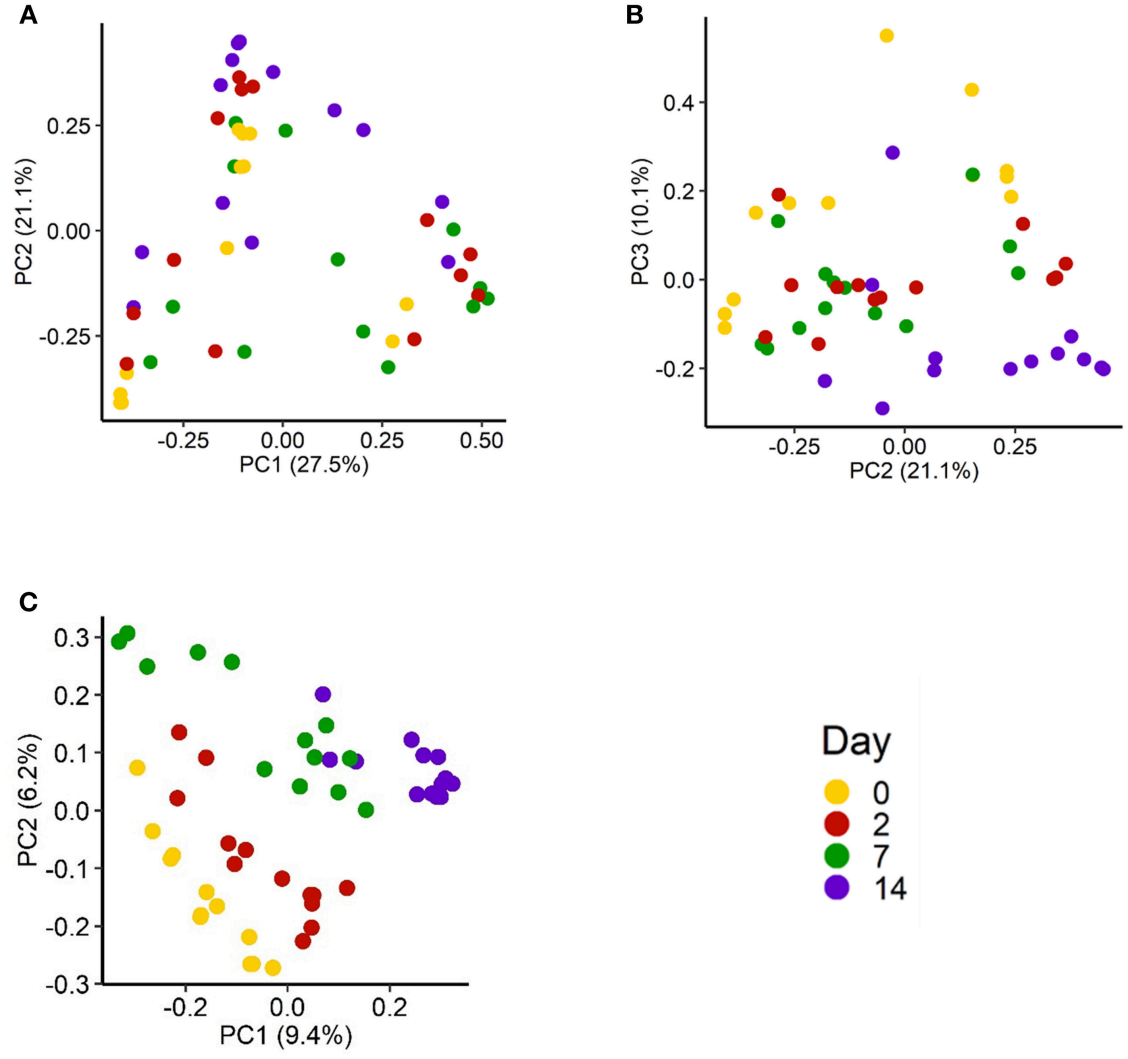
Principal coordinates analysis (PCoA) plot of the Bray-Curtis dissimilarities of the nasopharyngeal microbiota by sampling day for **(A)** PC1 vs. PC2 and **(B)** PC2 vs. PC3. **(C)** PCoA plot of the Jaccard distances of the nasopharyngeal microbiota of cattle (*n* = 13) by sampling day. The percentage of variation explained by each principal coordinate is indicated on the axes.

In total, 3,064 unique archaeal and bacterial OTUs were identified among the NP samples from 1,707,932 16S rRNA gene sequences. There was a noticeable shift in the microbial community structure of the nasopharynx as the cattle were transported to the auction market and then to the feedlot. When OTU abundance (Bray-Curtis dissimilarity) was taken into account the effect was weak but significant (Figures 1A,B; *R*^2^ = 0.17; *P* < 0.05). Among the different sampling times, day 0 and 14 NP samples were most dissimilar from each other (*R*^2^ = 0.18; *P* < 0.05). Only days 0 and 2 and days 2 and 7 were not significantly different (*R*^2^ <0.10; *P* > 0.05). We also assessed the temporal changes in the NP microbial community structure over time using the binary Jaccard distances (Figure 1C). This metric includes only the presence or absence of an OTU to compare microbial communities. Sampling time was observed to affect the community structure (*R*^2^ = 0.15; *P* < 0.05), again most strongly between day 0 and 14 NP samples (*R*^2^ = 0.15; *P* < 0.05).

### Longitudinal Changes Among the *Lactobacillales* Families

**Figure 2:**
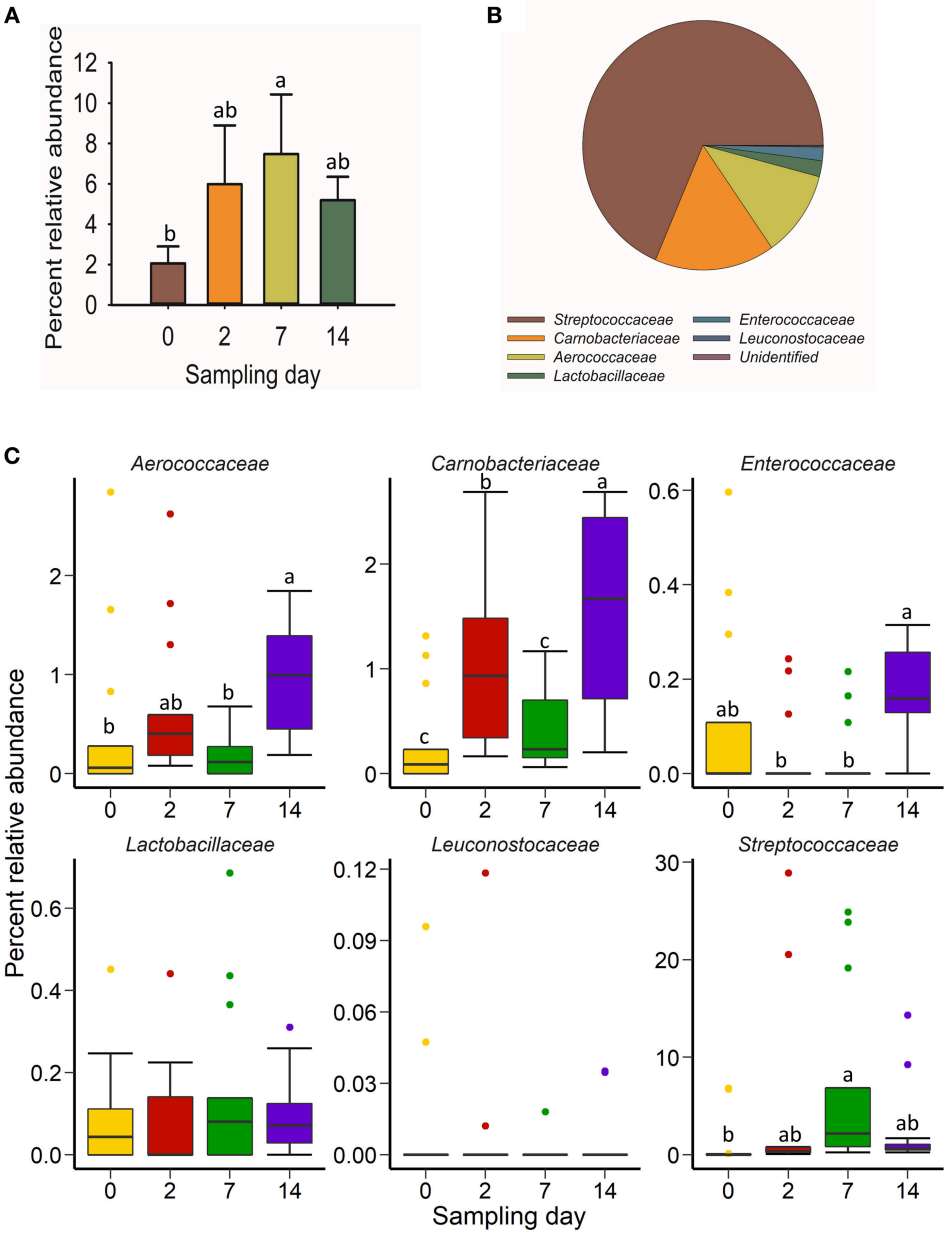
**(A)** Relative abundance of total lactic acid-producing bacteria (LAB) (defined as order *Lactobacillales*); **(B)** Proportion of LAB familes within the order of *Lactobacillales* across time points; **(C)** Box and whisker plots of the percent relative abundance of 6 families observed within LAB in the nasopharyngeal microbiota of cattle (*n* = 13) by sampling time. Colored dots indicate outliers. Different lowercase letters within each plot indicate significantly different means (*P* < 0.05).

We specifically analyzed the order *Lactobacillales*, which contains LAB. Overall, this order constituted 5.18% of the 16S rRNA gene sequences in the NP microbiota. The mean relative abundance of total LAB increased by 73% from the farm (day 0) to 5 days (day 7) after feedlot placement (*P* < 0.05), although this increase was temporary as the relative abundance of LAB at day 14 was not different from the level observed on day 2 (Figure 2A). Within *Lactobacillales*, all six families were detected from the steers during the course of the study (Figure 2B), including *Streptococcaceae* (3.55%), *Carnobacteriaceae* (0.84%), *Aerococcaceae* (0.58%), *Lactobacillaceae* (0.16%), *Enterococcaceae* (0.09%), and *Leuconostocaceae* (0.01%). There was an increase in the relative abundance of most of these LAB families over the course of the study (Figure 2C). For example, a significantly greater abundance of *Streptococcaceae* was observed on day 7 compared to day 0 (*P* > 0.05) and *Carnobacteriaceae* was enriched on day 2 and 14 compared to day 0 (*P* < 0.05), but not between day 7 and 0. The relative abundance of *Enterococcaceae* was lower on days 2 and 7 compared to day 0, but this family was enriched on day 14. Of note, due to higher variation between animals, no statistical difference in abundance of *Lactobacillaceae* was observed at any sampling times (*P* > 0.05). The relative abundance of *Leuconostocaceae*, the least abundant LAB family, was unchanged from day 0 through day 14.

### Longitudinal Changes Among the Archaeal and Bacterial Phyla and Genera in the Nasopharyngeal Microbiota

Across time and individual animals, a total of 26 different bacterial phyla were identified with *Proteobacteria* (36.1%), *Firmicutes* (20.1%), *Tenericutes* (19.3%), *Actinobacteria* (12.7%), and *Bacteroidetes* (8.6%) being the five most relatively abundant (data not shown). Overall, *Proteobacteria* remained the most relatively abundant phylum throughout the study. Interestingly, the relative abundance of most of these phyla varied significantly among individual animals on almost all sampling days (Figure S1). For example, the nasopharynx of two animals was largely colonized with members of the *Tenericutes* (>90%) on day 0.

**Figure 3:**
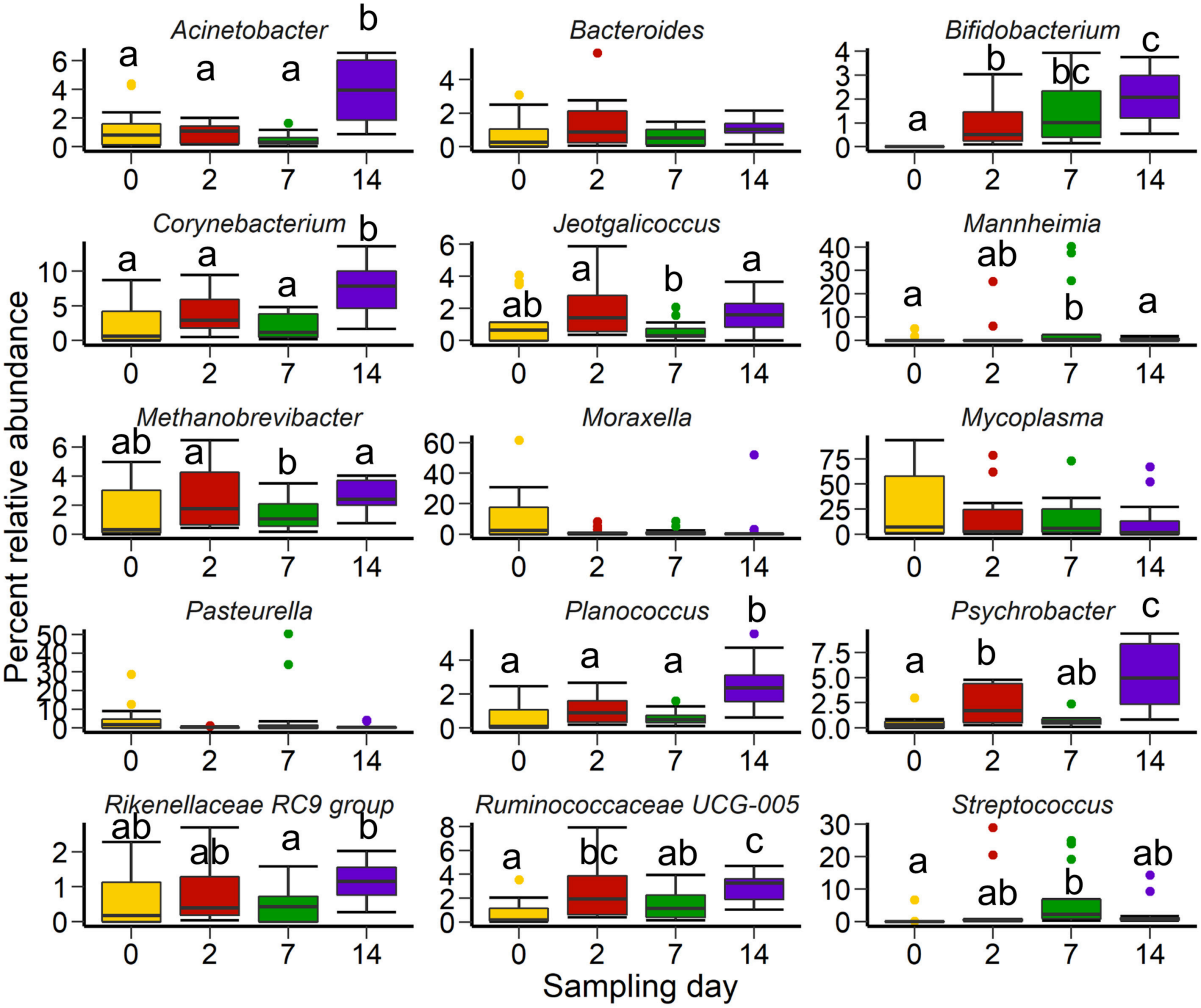
Box and whisker plots of the 15 most abundant genera in the nasopharyngeal microbiota of cattle (*n* = 13) by sampling time. Colored dots indicate outliers. The box in the box plots indicates the interquartile range (IQR) (middle 50% of the data), the middle line represents the median value, and the whiskers represents 1.5 times the IQR. Different lowercase letters indicate significantly different means (*P* < 0.05).

Of the 15 most relatively abundant genera, the relative abundance of *Bacteroides, Moraxella, Mycoplasma*, and *Pasteurella* did not differ by sampling time (Figure 3; *P* > 0.05). In contrast, *Acinetobacter, Corynebacterium*, and *Planococcus* were more relatively abundant on day 14 compared to all other sampling days (*P* < 0.05). Interestingly, *Bifidobacterium* was not detected in any animals on day 0 but became progressively more relatively abundant after feedlot placement and through to day 14. The relative abundance of both *Mannheimia* and *Streptococcus* was greater on days 2 and 7 compared to days 0 and 14 (*P* < 0.05). *Psychrobacter* spp. increased after feedlot entry and commingling at the auction market (day 2), although the relative abundance of this genus was variable during the experimental period. The relative abundance of the *Rikenellaceae* RC9 group was unchanged before and after feedlot placement but a lower relative abundance was detected on day 7 compared to day 14 (*P* < 0.05).

### Associations Between the 15 Most Relatively Abundant Genera

To identify potential associations among the 15 most relatively abundant NP bacteria, we used a stepwise-selected GLIMMIX model. As shown in Table 2, varying degrees of positive or negative associations between these genera were detected. *Moraxella, Corynebacterium, Methanobrevibacter*, and *Pasteurella* were negatively associated with *Mycoplasma (P* < 0.05), with *Methanobrevibacter* spp. associated with the most negative regression coefficient (*b*_ME_ = −0.676)*. Planococcus* was found to be the only genus that was significantly associated with *Mannheimia*. The odds ratio estimate revealed that a one unit increase in *Planococcus* could decrease the relative abundance of *Mannheimia* by 0.9 units (data not shown). The relative abundance of *Pasteurella* was predicted to be negatively affected by the presence of *Psychrobacter*, but positively affected by *Bifidobacterium*.

**Table 2:** Associations among the 15 most relatively abundant genera in the nasopharyngeal microbiota of cattle (*n* = 13) across time^a^.

**Y**	***b*_**0**_**	***b*_**time**_**	$b_{\text{time}}^{2}$	**Mo**	**My**	**Co**	**Bi**	**Ma**	**Ps**	**Me**	**Ru**	**Ac**	**Je**	**Pl**	**Ba**	**Ri**	**St**	**Pa**	**Interactions**									
																			Ps^*^Ac	Me^*^Ma	My^*^Ru	Je^*^My	Pl^*^Me	Ac^*^Ri	**Je × Pl**	Time^*^Pl	Time^*^Ba	Ps^*^Je
Mo	0.56	–	–	–	0.562	–	–	–	–	−0.045	–	–	–	–	–	–	–	–	–	–	–	–	–	–	–	–	–	–
My	1.63	–	–	−0.068	–	−0.171		–	–	−0.676	–	–	–	–	–	–	–	−0.045	–	–	–		–	–	–	–	–	–
Co	−4.61	–	–	–	–	–	–	–	0.287		–	0.358	0.159	–	–	–	–	–	−0.047	–	–	–	–	–	–	–	–	–
Bi	−7.32	0.529	−0.027	–	–	–	–	−0.040	–	−0.095	0.356	–	0.232	–	–	–	–	0.011	–	0.029	–	–		–	–	–	–	
Ma	−1.28	–	–	–	–	–	–	–	–	–	–	–	–	−2.255	–	–	–	–	–	–	–	–	–	–	–	–	–	–
Ps	−6.50	0.063	–	–	0.003	0.157	–	–	–	0.322	0.212		−0.053	0.284	–	–	–	–	–	–	−0.009	0.015	−0.12	–	–	–	–	–
Me	−5.47	–	–	–	–	–	0.145	–	–	–	–	0.356	0.437	0.132	–	0.659	–	–	–	–	–	–	–	−0.197	−0.238	–	–	–
Ru	−5.91	0.096	–	–	–	–	0.214	–	–	0.213	–	–	–	0.767	–	–	−0.022	–	–	–	–		–	–	–	−0.055	–	–
Ac	−5.30	–	–	–	–	0.213	–	–	–	–	–	–	–	–	–	–	–	–	–	–		–	–	–	–	–	–	–
Je	−6.02	–	–	–	–	0.154	–	–	–	–	–	–	–	–	–	–	–	–	–	–		–	–	–	–	–	–	
Pl	−6.31	0.109	–	–	–	–	–	–	0.106	–	–	0.169	0.388	–	0.238	–	–	–	–	–	–	–	–	–	–	–	−0.038	−0.045
Ba	−5.75	–	–	–	–	–	–	–	–	0.272	0.156	–	–	–	–	–	–	–	–	–	–	–	–	–	–	–	–	–
Ri	−5.98	0.030	–	–	–	–	–	–	–	0.372	–	–	–	–	–	–	–	–	–	–	–	–	–	–	–	–	–	–
St	−2.98	0.172	–	–	−0.055	–	–	−0.038	–	–	–	−0.882	–	–	–	–	–	–	–		–	–		–	–	–	–	–
Pa	−3.42	–	–	–	–	–	0.900	–	−0.751	–	–	–	–	–	–	–	–	–	–	–	–		–	–		–	–	–

a *Associations were measured using the following stepwise-selected Generalized Liner Mixed Model: logit (Ŷ) = ln (π/1 – π) = b_0_ + b_time_ + b_time2_ + b_1_ (X_1_) + … + b_n_ (X_n_). The values were the mean estimated regression coefficients. The negative and positive values represent the negative association (mutual-exclusion) or positive association (co-occupation), respectively, between the genera predicted and the independent variables (genus, time, and interactions). Only those independent variables that had significant effects (P < 0.05) on the predicted genus remained in the model and are presented in this table. Ac, Acinetobacter; Ba, Bacteroides; Bi, Bifidobacterium; Co, Corynebacterium; Je, Jeotgalicoccus; Ma, Mannheimia; Me, Methanobrevibacter; Mo, Moraxella; My, Mycoplasma; Pa, Pasteurella; Pl, Planococcus; Ps, Psychrobacter; Ri, Rikenellaceae RC9 gut group; Ru, Ruminococcaceae UCG-005; St, Streptococcus*.

Members of the *Psychrobacter, Acinetobacter*, and *Jeotgalicoccus* genera were positively associated with *Corynebacterium* and *Planococcus*. The relative abundance of *Streptococcus* spp. were found to be negatively affected by *Mycoplasma, Mannheimia*, and *Acinetobacter* spp. Among the 15 most relatively abundant genera, *Bifidobacterium, Psychrobacter, Ruminococcaceae UCG-005*, and *Planococcus* were predicted to be the most interactive genera whose relative abundance were significantly influenced by the presence of four or more different genera. A positive association of sampling time with the relative abundance of these four genera was detected.

We also observed a significant interaction effect among multiple genera (Table 2). For example, although both *Psychrobacter* and *Acinetobacter* had a positive association with *Corynebacterium*, when analyzed individually, the interaction of these two genera (co-existence) was negatively associated with *Corynebacterium*. The interactions between *Mycoplasma* and *Ruminococcaceae*, or *Planococcus* and *Methanobrevibacter* displayed a negative association with *Psychrobacter*, whereas, the interaction of *Mycoplasma* and *Jeotgalicoccus* were positively associated with *Psychrobacter*.

### Differentially Abundant Taxa in the Nasopharyngeal Microbiota Following Feedlot Placement

**Figure 4:**
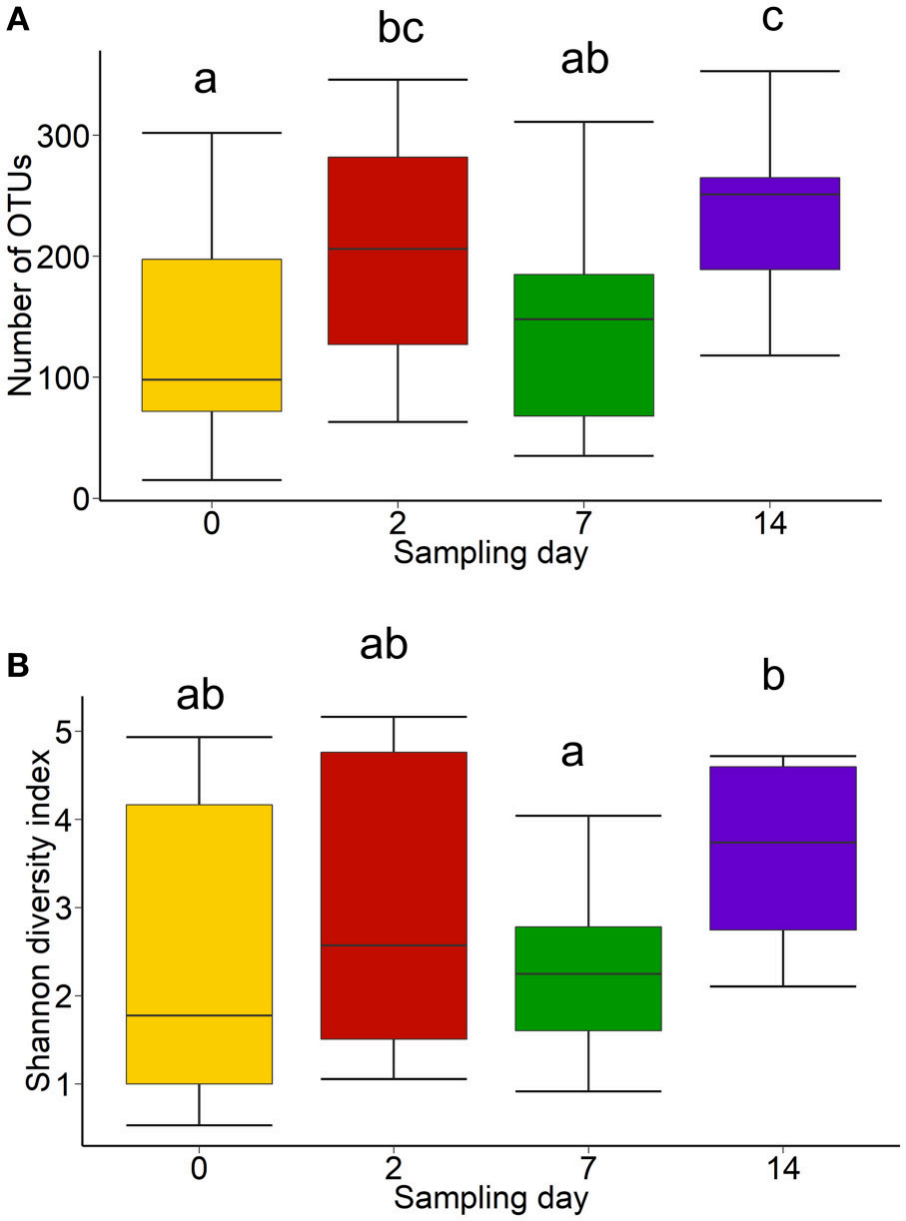
Box and whisker plots of the **(A)** number of OTUs and **(B)** Shannon diversity index of the nasopharyngeal microbiota of cattle (*n* = 13) by sampling time. The box in the box plots indicates the interquartile range (IQR) (middle 50% of the data), the middle line represents the median value, and the whiskers represents 1.5 times the IQR. Different lowercase letters indicate significantly different means (*P* < 0.05).

In addition to the changes within the 15 most relatively abundant genera discussed above, there was more microbial richness (number of OTUs) in the NP microbiota at day 14 compared with day 0, although diversity (Shannon diversity index) was unaffected due to the variability in the day 0 samples (Figure 4). Because the day 0 and 14 NP samples were most dissimilar based on both Bray-Curtis dissimilarities and Jaccard distances, we identified which OTUs were most differentially abundant between these sampling times. There was a total of 121 OTUs that were differentially abundant between day 0 and 14 with 105 of these OTUs enriched on day 14, having log_2_ fold changes ranging from 1.6 to 10.3 (Table S1). OTUs classified as *Acinetobacter, Bifidobacterium, Corynebacterium, Mannheimia, Planococcus, Prevotella*, and *Psychrobacter* were among the abundant OTUs that were enriched in the day 14 NP microbiota. Notably, only 16 OTUs were more abundant in the day 0 NP samples. However, these did include the abundant *Mycoplasma, Moraxella*, and *Pasteurella* OTUs. Differentially abundant OTUs with a log_2_ fold change of at least ± 6 are shown in Table 3.

**Table 3:** Differentially abundant OTUs in the nasopharyngeal microbiota of feedlot cattle between day 0 and 14 (*n* = 13)^a^.

**OTU ID**	**Mean abundance**	**log_**2**_ fold change^b^**	**FDR^c^**	**Phylum**	**Family**	**Genus**
OTU66	181.4	−10.3	4.66E-57	*Actinobacteria*	*Bifidobacteriaceae*	*Bifidobacterium*
OTU496	77.8	−9.1	1.98E-18	*Bacteroidetes*	*Prevotellaceae*	*Prevotella*
OTU2836	68.8	−8.8	0.000281	*Proteobacteria*	*Pasteurellaceae*	*Mannheimia*
OTU2280	59.5	−8.7	1.56E-37	*Firmicutes*	*Ruminococcaceae*	*Ruminococcaceae UCG-013*
OTU229	48.9	−8.4	1.01E-27	*Actinobacteria*	*Pseudonocardiaceae*	*Saccharopolyspora*
OTU1569	45.1	−8.3	1.43E-20	*Firmicutes*	*Lachnospiraceae*	*Lachnospiraceae NK4A136 group*
OTU2711	44.1	−8.3	8.15E-11	*Proteobacteria*	*Succinivibrionaceae*	*Ruminobacter*
OTU3	39.5	−8.1	4.91E-06	*Euryarchaeota*	*Methanobacteriaceae*	*Methanobrevibacter*
OTU1108	28.8	−7.6	2.14E-08	*Firmicutes*	*Aerococcaceae*	*Facklamia*
OTU511	27.3	−7.6	6.27E-27	*Bacteroidetes*	*Prevotellaceae*	*Prevotella*
OTU1628	27.0	−7.6	1.02E-25	Firmicutes	*Lachnospiraceae*	NA
OTU1553	25.3	−7.5	0.000108	*Firmicutes*	*Lachnospiraceae*	*Lachnospiraceae NK3A20 group*
OTU1457	25.0	−7.4	1.83E-27	*Firmicutes*	*Lachnospiraceae*	*Acetitomaculum*
OTU1091	25.0	−7.4	1.99E-05	*Firmicutes*	*Staphylococcaceae*	*Staphylococcus*
OTU1519	24.8	−7.4	8.64E-10	*Firmicutes*	*Lachnospiraceae*	*Coprococcus*
OTU96	23.5	−7.4	1.39E-09	*Actinobacteria*	*Dietziaceae*	*Dietzia*
OTU2871	22.7	−7.3	4.21E-12	*Proteobacteria*	*Moraxellaceae*	*Psychrobacter*
OTU137	21.5	−7.2	2.63E-06	*Actinobacteria*	*Intrasporangiaceae*	*Janibacter*
OTU1107	21.3	−7.2	0.00016	*Firmicutes*	*Aerococcaceae*	*Facklamia*
OTU144	21.1	−7.2	1.68E-07	*Actinobacteria*	*Intrasporangiaceae*	*Ornithinimicrobium*
OTU642	20.4	−7.2	7.59E-09	*Bacteroidetes*	*Rikenellaceae*	*Rikenellaceae RC9 gut group*
OTU2450	19.6	−7.1	4.39E-11	*Firmicutes*	*Veillonellaceae*	*Anaerovibrio*
OTU2384	18.8	−7.0	5.02E-11	*Firmicutes*	*Ruminococcaceae*	*Ruminococcus*
OTU77	18.2	−7.0	1.90E-06	*Actinobacteria*	*Corynebacteriaceae*	*Corynebacterium*
OTU1280	15.5	−6.8	8.27E-10	*Firmicutes*	*Clostridiaceae*	*Clostridium sensu stricto*
OTU1052	15.3	−6.7	1.67E-05	*Firmicutes*	*Planococcaceae*	*Lysinibacillus*
OTU2715	14.3	−6.6	2.77E-19	*Proteobacteria*	*Succinivibrionaceae*	*Succinivibrio*
OTU644	14.1	−6.6	2.59E-05	*Bacteroidetes*	*Rikenellaceae*	*Rikenellaceae RC9 gut group*
OTU1484	14.0	−6.6	5.07E-10	*Firmicutes*	*Lachnospiraceae*	*Blautia*
OTU1629	13.5	−6.6	1.52E-12	Firmicutes	*Lachnospiraceae*	NA
OTU225	13.5	−6.6	3.45E-07	*Actinobacteria*	*Pseudonocardiaceae*	*Prauserella*
OTU1176	37.8	−6.5	2.91E-11	*Firmicutes*	*Streptococcaceae*	*Streptococcus*
OTU1570	13.4	−6.5	0.00019	*Firmicutes*	*Lachnospiraceae*	*Lachnospiraceae NK4A136 group*
OTU1143	12.9	−6.5	2.44E-06	*Firmicutes*	*Enterococcaceae*	*Enterococcus*
OTU345	12.8	−6.5	2.04E-09	Bacteroidetes	*Barnesiellaceae*	NA
OTU497	12.8	−6.5	5.74E-05	*Bacteroidetes*	*Prevotellaceae*	*Prevotella*
OTU1469	12.6	−6.5	3.86E-05	*Firmicutes*	*Lachnospiraceae*	*Agathobacter*
OTU646	12.0	−6.4	5.70E-06	*Bacteroidetes*	*Rikenellaceae*	*Rikenellaceae RC9 gut group*
OTU1304	11.7	−6.4	5.69E-06	*Firmicutes*	*Clostridiaceae*	*Proteiniclasticum*
OTU2939	11.5	−6.3	1.11E-05	Spirochaetes	*Spirochaetaceae*	*Treponema*
OTU1137	11.4	−6.3	2.39E-07	*Firmicutes*	*Carnobacteriaceae*	*Desemzia*
OTU830	11.0	−6.3	8.86E-06	*Bacteroidetes*	*Weeksellaceae*	*Chishuiella*
OTU2451	10.8	−6.2	6.82E-06	*Firmicutes*	*Veillonellaceae*	*Anaerovibrio*
OTU2938	10.8	−6.2	1.11E-06	Spirochaetes	*Spirochaetaceae*	*Treponema*
OTU224	10.5	−6.2	1.07E-10	Actinobacteria	*Pseudonocardiaceae*	NA
OTU2868	380.6	−6.2	3.82E-12	*Proteobacteria*	*Moraxellaceae*	*Psychrobacter*
OTU185	10.1	−6.1	0.000464	Actinobacteria	*Micrococcaceae*	NA
OTU1473	9.9	−6.1	2.62E-05	*Firmicutes*	*Lachnospiraceae*	*Anaerosporobacter*
OTU379	9.8	−6.1	7.48E-07	Bacteroidetes	*Muribaculaceae*	NA
OTU1312	9.3	−6.0	5.29E-08	Firmicutes	*Clostridiales_vadinBB60_group*	NA

a *Only those OTUs with a log_2_ fold change of at least ± 6 are displayed. Mean abundance values are the mean abundance for each OTU among all day 0 and 14 NP samples*.

b *Negative fold change values indicate OTUs that were enriched in the day 14 NP samples*.

c *FDR, false discovery rate*.

We also compared the day 0 and 2 NP samples to identify OTUs in the NP microbiota that were altered after transport to and from the auction market, as there was a significant increase in microbial richness (number of OTUs) during this short period. In total, 25 OTUs were differentially abundant between day 0 and 2, with 21 OTUs more abundant on day 2 and 4 on day 0 (Table 4). *Bifidobacterium, Psychrobacter*, and *Streptococcus* were among the most enriched genera on day 2 while *Pasteurella* and *Sphingomonas* OTUs were more abundant on day 0 prior to transport to the auction market.

**Table 4:** Differentially abundant OTUs in the nasopharyngeal microbiota of feedlot cattle between day 0 and 2 (*n* = 13)^a^.

**OTU ID**	**Mean**	**log_**2**_ (fold change)**	**FDR^**c**^**	**Phylum**	**Family**	**Genus**
OTU66	52.2	−8.8^b^	1.33E-22	*Actinobacteria*	*Bifidobacteriaceae*	*Bifidobacterium*
OTU1107	29.4	−7.9	4.79E-10	*Firmicutes*	*Aerococcaceae*	*Facklamia*
OTU67	23.2	−7.6	0.00014576	*Actinobacteria*	*Bifidobacteriaceae*	*Bifidobacterium*
OTU1553	22.5	−7.5	0.000198397	*Firmicutes*	*Lachnospiraceae*	*Lachnospiraceae NK3A20 group*
OTU1280	10.6	−6.4	0.000751528	*Firmicutes*	*Clostridiaceae*	*Clostridium sensu stricto*
OTU246	10.2	−6.4	0.002568228	*Actinobacteria*	*Atopobiaceae*	*Olsenella*
OTU1176	24.0	−6.2	5.08E-07	*Firmicutes*	*Streptococcaceae*	*Streptococcus*
OTU1398	8.5	−6.1	0.020739752	*Firmicutes*	*Family_XIII*	*Family XIII AD3011 group*
OTU1069	8.4	−6.1	0.020739752	*Firmicutes*	*Planococcaceae*	*Sporosarcina*
OTU2938	7.3	−5.9	0.002568228	Spirochaetes	*Spirochaetaceae*	*Treponema*
OTU1434	6.5	−5.7	0.02991382	Firmicutes	*Family_XIII*	NA
OTU1126	5.4	−5.5	0.035196373	*Firmicutes*	*Carnobacteriaceae*	*lloiococcus*
OTU2715	4.2	−5.1	0.032009852	*Proteobacteria*	*Succinivibrionaceae*	*Succinivibrio*
OTU1840	18.8	−4.8	0.000909354	*Firmicutes*	*Peptostreptococcaceae*	*Clostridioides*
OTU2868	121.4	−4.7	5.08E-07	*Proteobacteria*	*Moraxellaceae*	*Psychrobacter*
OTU1129	26.6	−4.6	0.000372247	*Firmicutes*	*Carnobacteriaceae*	*Atopostipes*
OTU2385	11.7	−4.5	0.028989764	*Firmicutes*	*Ruminococcaceae*	*Ruminococcus*
OTU2029	5.7	−4.5	0.029881416	*Firmicutes*	*Ruminococcaceae*	*Ruminococcaceae NK4A214 group*
OTU2869	35.3	−3.9	0.01341607	*Proteobacteria*	*Moraxellaceae*	*Psychrobacter*
OTU1842	21.7	−3.1	0.02991382	*Firmicutes*	*Peptostreptococcaceae*	*Paeniclostridium*
OTU1139	58.1	−3.0	0.006904558	*Firmicutes*	*Carnobacteriaceae*	*Jeotgalibaca*
OTU1016	217.3	1.5	0.0154763	Firmicutes	*Bacillaceae*	NA
OTU2673	138.3	1.8	0.009752217	*Proteobacteria*	*Sphingomonadaceae*	*Sphingomonas*
OTU2911	147.4	1.9	0.002568228	Proteobacteria	*Rhodanobacteraceae*	NA
OTU2839	611.9	4.0	0.009752217	*Proteobacteria*	*Pasteurellaceae*	*Pasteurella*

a *Mean abundance values are the mean abundance for each OTU among all day 0 and 2 NP samples*.

b *Negative fold change values indicate OTUs that were enriched in the day 2 NP samples whereas positive fold values show OTUs that were reduced in day 2 NP samples*.

c *FDR, false discovery rate*.

### The Relationship Between LAB and BRD-Associated *Pasteurellaceae* Family Members

There were positive Spearman correlations between all LAB families, with the exception of an inverse association between *Streptococcaceae* and *Leuconostocaceae* (Table 5). Positive correlations were statistically significant between: *Aerococcaceae, Carnobacteriaceae, Enterococcaceae*, and *Lactobacillaceae*; and *Leuconostocaceae, Aerococcaceae*, and *Carnobacteriaceae*; and between *Streptococcaceae* and *Carnobacteriaceae* (*P* < 0.05). Although relative abundance of all the observed LAB families was inversely correlated with the *Pasteurellaceae* family, only the correlation between *Enterococcaceae* and *Pasteurellaceae* was statistically significant (*P* = 0.05).

**Table 5:** Correlations between families within the order *Lactobacillales*, and the BRD-associated *Pasteurellaceae* family in the nasopharyngeal microbiota of cattle (*n* = 13) across sampling times.

	**Time**	***Aerococcaceae***	***Carnobacteriaceae***	***Enterococcaceae***	***Lactobacillaceae***	***Leuconostocaceae***	***Streptococcaceae***	***Pasteurellaceae***
Time	**1.000**	**0.318**^**a**^	**0.476**	**0.214**	**0.101**	–**0.039**	**0.534**	–**0.097**
		0.02^b^	0.0004	0.13	0.48	0.78	<0.0001	0.49
*Aerococcaceae*	**0.318**	**1.000**	**0.792**	**0.366**	**0.479**	**0.334**	**0.043**	–**0.069**
	0.022		<0.0001	0.008	0.0003	0.01	0.76	0.62
*Carnobacteriaceae*	**0.476**	**0.792**	**1.000**	**0.531**	**0.508**	**0.278**	**0.274**	–**0.212**
	0.0004	<0.0001		<0.0001	0.0001	0.04	0.05	0.13
*Enterococcaceae*	**0.214**	**0.366**	**0.531**	**1.000**	**0.366**	**0.200**	**0.019**	–**0.267**
	0.12	0.008	<0.0001		0.008	0.15	0.89	0.05
*Lactobacillaceae*	**0.101**	**0.479**	**0.508**	**0.366**	**1.000**	**0.242**	**0.153**	–**0.084**
	0.47	0.0003	0.0001	0.008		0.08	0.27	0.55
*Leuconostocaceae*	–**0.039**	**0.334**	**0.278**	**0.200**	**0.242**	**1.000**	–**0.073**	–**0.125**
	0.78	0.01	0.04	0.154	0.08		0.60	0.37
*Streptococcaceae*	**0.534**	**0.043**	**0.274**	**0.019**	**0.153**	–**0.073**	**1.000**	–**0.057**
	<0.0001	0.76	0.05	0.89	0.27	0.60		0.68
*Pasteurellaceae*	–**0.097**	–**0.069**	–**0.212**	–**0.267**	–**0.084**	–**0.125**	–**0.057**	**1.000**
	0.49	0.62	0.13	0.05	0.55	0.37	0.68	

a Spearman's rank correlation coefficient. Positive coefficient values indicate a positive correlation between two variable means; negative coefficient values indicate a negative correlation between the two variable means shown in bold.

b *P-value*.

The dynamic changes in the relationship between LAB families and *Pasteurellaceae* over time are shown in Figure S2. *Enterococcaceae* was observed by a over time negative correlation with *Pasteurellaceae* at all sampling points. The correlation between *Lactobacillaceae* and *Pasteurellaceae* became increasingly negative. Although a weak negative correlation between *Streptococcaceae* and *Pasteurellaceae* was observed on day 0, the correlation shifted after feedlot placement, becoming more positive over time. The remaining LAB families exhibited inconsistent correlations with *Pasteurellaceae* at different sampling points, including after feedlot placement.

**Figure 5:**
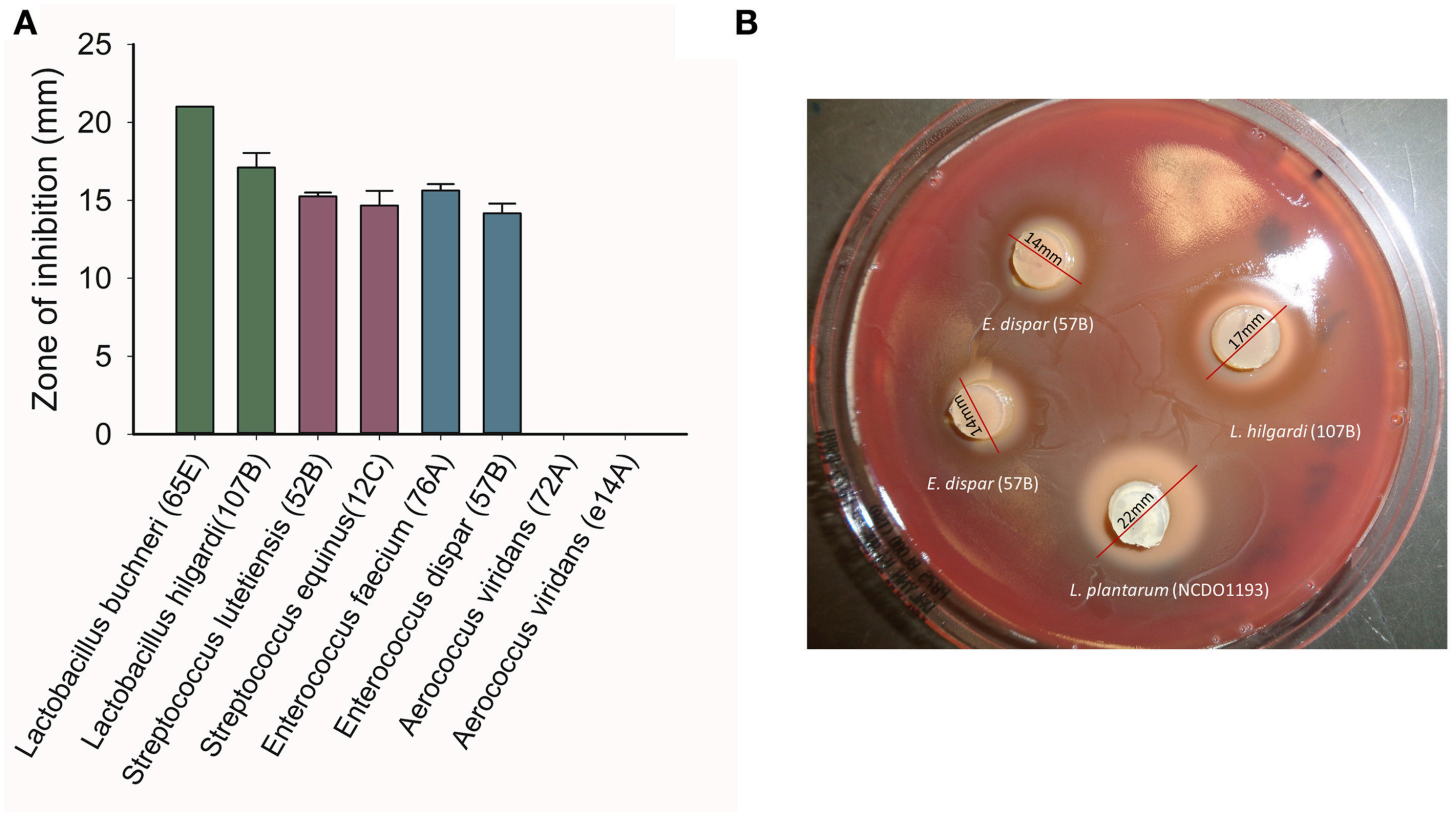
Growth inhibitory effects of lactic acid bacteria (LAB) strains isolated from the nasopharynx of healthy feedlot cattle against a bovine respiratory pathogen *Mannheimia haemolytica* serotype 1 strain, as determined by the agar slab method. **(A)** mean inhibitory zones from triplicate tests. **(B)** An example of the agar slabs showing inhibition zones on a lawn of *M. haemolytica*.

Because of the negative associations observed for LAB families and *Pasteurellaceae*, we tested whether members of LAB families, isolated from beef cattle, were capable of inhibiting the BRD-associated pathogen *M. haemolytica*. All tested isolates within the genera of *Lactobacillus, Streptococcus* and *Enterococcus* were able to inhibit the growth of *M. haemolytica*, with inhibition zones ranging from 14 to 21 mm (Figure 5). Among these inhibitory isolates, *Lactobacillus* strains exhibited greater inhibition of *M. haemolytica* (zones of inhibition ≥ 17 mm) while moderate inhibition of *M. haemolytica* was observed with *Streptococcus* and *Enterococcus* isolates. However, none of the *Aerococcus* isolates inhibited *M. haemolytica*.

## Discussion

Increasing evidence suggests that the microbiota of the respiratory tract plays an important role in maintaining respiratory health of cattle. Additionally, it may also be a source of antimicrobials that inhibit respiratory pathogens in feedlot cattle (Amat et al., 2016, 2018; Timsit et al., 2016a). In North America, beef calves can be shipped to auction markets where they are held and mixed with other sources of cattle before transported to the feedlots. While auction market-derived calves have been associated with increased risk of BRD, few studies have actually tested such an association at the microbiological level (Taylor et al., 2010). Previously, we investigated the effect of transporting cattle from a closed herd directly to the feedlot (Holman et al., 2017). In the current study, we transported cattle from this same herd but included a 48 h stay at an auction market before shipping to the feedlot.

### The Prevalence of Cultured BRD-Associated Pathogens

Almost all calves remained culture-negative for *M. haemolytica* and *H. somni* during the course of the study, and those that were positive for *P. multocida* on the farm were negative after feedlot placement, despite the fact that they were commingling at an auction market with other sources of cattle for 48 h prior to feedlot placement. Some studies have suggested that commingling or mixing with different sources of cattle in the auction market increases the risk of BRD pathogen exposure and BRD in feedlot cattle. Ribble et al. (1995) evaluated the correlation of commingling with the development of BRD in calves following feedlot placement using a large number of cattle. They concluded that commingling increased the risk of BRD in the calves following feedlot placement. Step et al. (2008) also observed significantly greater total mortality due to BRD in auction market-derived calves compared to calves purchased directly from a ranch. In our study, the lack of NP colonization by BRD-associated pathogens during the course of mixing with other calves at the auction market indicated that spread of pathogenic bacteria at the auction market was limited. This was also shown by Stroebel et al. (2018) who observed no effect of commingling at an auction market for 24 h on the prevalence of BRD-associated families *Mycoplasmataceae* and *Pasteurellaceae* in recently weaned beef heifers. However, because bacterial colonization of other commingled calves at the auction market was not evaluated, it is possible that spread of pathogens was limited due to these commingled calves not being colonized by BRD pathogens themselves.

Step et al. (2008) also compared calves that were simultaneously weaned and shipped to a research feedlot to those that were weaned and held on a ranch for 45 days prior to being transported. The total morbidity associated with BRD was significantly lower in calves weaned 45 days before transport to the feedlot than calves weaned and immediately transported to the feedlot (35 vs. 6%). Although a relatively small number of cattle were followed in our study, all animals remained healthy and there was no proliferation of specific BRD-associated bacterial pathogens observed by either culturing or 16S rRNA gene sequencing. While this may indicate that commingling had a limited effect on bacterial transfer at the auction market, it is important to note that viral agents were not evaluated. In addition, the results may also be due to the weaning history and the origin of the herd the calves originated from, as well as the nature of their nasopharyngeal microbiota. In the current study, the calves were sourced from a disease-free herd that was closed to cattle sourced from outside the herd, limiting introduction of BRD-associated bacterial and viral pathogens prior to shipment. They were also weaned 41 days prior to shipment to the auction market and therefore were expected to have enhanced immunity to cope with stress during transportation to and commingling at the auction market, handling, and viral infection. Prior to shipping, these calves would have also experienced lower levels of stress, particularly stress associated with maternal-separation and eating from a bunk. It is therefore assumed that their risk of developing BRD was low and that they were likely less susceptible to colonization by BRD-associated pathogens. These results are also in agreement with our earlier findings with cattle from the same farm that were directly transported to the feedlot. In that study, we were able to isolate only *P*. *multocida* from the nasopharynx and there was no increase in prevalence during the same 14 days period (Holman et al., 2017). Overall, the data suggested that transfer of bacterial pathogens at the auction market was limited in this group of low-stress calves.

### Effect of Transport and Auction Market Commingling on the Nasopharyngeal Microbiota

In agreement with our study, *Proteobacteria, Firmicutes, Tenericutes, Actinobacteria, Bacteroidetes*, and *Firmicutes* have been reported to be the most relatively abundant phyla in nasopharynx of early, or newly weaned, and feedlot placed cattle (Timsit et al., 2016b; Holman et al., 2017; Stroebel et al., 2018). Nevertheless, the proportion of some of these phyla observed in the present study was different from that of some other studies. The relative abundance of *Firmicutes* in the nasopharynx of newly weaned and auction or cow-calf ranch derived feedlot heifers was reported by Stroebel et al. (2018) to be only 3% compared with 20.1% in our study. The third most relatively abundant phylum that we observed was *Tenericutes* (19%), and this phylum has been reported to be the most abundant phylum in the nasopharynx (Stroebel et al., 2018; Timsit et al., 2018) and trachea (Timsit et al., 2018) of feedlot cattle sometimes comprising more than 40% of the total microbial community.

Overall, the structure and composition of the NP microbial community shifted after transport to the auction market and then to the feedlot. In an earlier study, we transported beef cattle heifers from the same farm directly to the same feedlot and noted a similar change in the community membership following the first 2 weeks of feedlot placement (Holman et al., 2017).

The increase in microbial diversity of the NP microbiota after feedlot placement might be due to several factors. It is possible that the feedlot was a source of bacteria that colonized the respiratory tract through aspiration, or airborne nutrients that may have promoted growth of certain bacterial species. In addition, the change in diet may have altered the gastrointestinal bacterial microbiota, which may also colonize the respiratory tract through oropharyngeal transfer (Hall et al., 2017). As mentioned above, the cattle used in our study were expected to be immunocompetent, although it cannot be ruled out that some alteration in immunity occurred after transportation, and that this potentially affected host modulation of the respiratory microbiome (Hooper et al., 2012). Furthermore, potential networks of co-occurrence or co-exclusion associations predicted among the 15 most relatively abundant genera suggested that the NP microbial community membership before or after feedlot arrival may be important factors in the susceptibility of the nasopharynx to colonization by novel bacterial species.

A large number of OTUs were increased in abundance as the cattle moved from the farm at day 0 through to day 14 when they had passed through the auction market and had been in the feedlot for 12 days. Among these OTUs, the increase in *Bifidobacterium* was particularly striking given the complete absence of this genus in the day 0 samples. This suggests that at least one species in this genus colonized as a result of environmental exposure, contact with other cattle, or stress-related factors. While typically associated with the gastrointestinal tract of mammals, *Bifidobacterium* has repeatedly been identified in the respiratory tract of cattle (Holman et al., 2015a, 2017). Thus, digesta of the gastrointestinal (GI) tract, or feces and manure, may be a source of this genus. In support of this, the relative abundance of *Bifidobacterium* spp. after feedlot entry was associated with an increased relative abundance of *Ruminococcaceae* UCG-005, a taxon also associated with the GI tract. Similar to many of the LAB, certain *Bifidobacterium* spp. are used as probiotic agents and have been investigated in humans to prevent or treat upper respiratory tract infections (Popova et al., 2012). Whether *Bifidobacterium* spp. in the upper respiratory tract of cattle confers a similar beneficial effect is unknown. However, given the strong positive correlation between the *Bifidobacterium* and *Pasteurella* genera in the present study, future research should focus on assessing whether the presence of *Bifidobacterium* spp. increases colonization and proliferation of *Pasteurella* in nasopharynx of feedlot cattle.

*Prevotella* spp. are obligate anaerobes that have been identified from both nasopharynx and tracheal samples obtained from cattle, albeit at a lower relative abundance (Lima et al., 2016; Timsit et al., 2018; Klima et al., 2019). The relative abundance of this genus in the nasopharynx has been reported to be similar among healthy or pneumonic dairy calves (Lima et al., 2016). Tracheal samples from healthy feedlot cattle have also been noted to be enriched with *Prevotella* compared to those with BRD (Timsit et al., 2017). However, the potential role of this genus in the respiratory tract of cattle remains unknown. *Prevotella* is typically the most abundant genus in the rumen (Henderson et al., 2015), and *Prevotella* spp. respond to the acidity of the rumen, becoming more enriched when a grain-based diet is fed to the cattle (Petri et al., 2013). It is therefore plausible that the increased abundance of *Prevotella* in the present study may be due to the changes in the diet after feedlot placement.

Other abundant OTUs belonging to the *Acinetobacter, Psychrobacter*, and *Corynebacterium* genera also increased in relative abundance from day 0 to day 14. The positive associations observed among these three genera indicate that they coexist in the nasopharynx and may mutually benefit each other. These genera are often reported to be dominant in the nasopharynx of healthy feedlot cattle (Holman et al., 2017, 2018; Zeineldin et al., 2017; Stroebel et al., 2018) and so this microbial state may reflect one that is more mature and stable in feedlots. *Planococcus* spp. were also significantly enriched during the first 12 days of feedlot placement. This was the only genus that had a strong inverse association with *Mannheimia*. This bacterial genus has not been well-characterized but it warrants further research given the importance of *Mannheimia* spp. in the development of BRD.

Interestingly, we also observed a decrease in the abundance of OTUs assigned to the *Moraxella, Mycoplasma*, and *Pasteurella* genera from day 0 to 14. One of the possible factors contributing to the reduction of *Pasteurella* spp. after feedlot placement might be the increased relative abundance of the commensal bacterial genus *Psychrobacter*, which displayed a strong negative association with *Pasteurella*. Based on the strong association of members of the *Mycoplasma* and *Moraxella*, it is plausible to infer that reduced *Mycoplasma* abundance may have resulted in the decrease of *Moraxella* if the existence of *Moraxella* depends on *Mycoplasma*. While several studies have reported an increase in nasopharyngeal colonization after feedlot placement (McMullen et al., 2018; Stroebel et al., 2018; Holman and Alexander, 2019), our study only lasted 14 days, thus changes in the microbiota may have occurred subsequent to the last sampling. In contrast to *Moraxella*, the relative abundance of *Corynebacterium* and *Methanobrevibacter* was inversely associated with *Mycoplasma*. Therefore, the enrichment of these commensal genera, and perhaps also the increased diversity of the microbiota after feedlot placement, may have resulted in an undesirable nutrient landscape for *M. bovis*, resulting in a reduction in the relative abundance of *Mycoplasma*.

### Relative Abundance and Antimicrobial Activity of Lactic Acid-Producing Bacteria and Correlation With the BRD-Associated *Pasteurellaceae* Family

To the authors' knowledge, this is the first study to report specifically on the longitudinal changes in the relative abundance of LAB bacteria, defined as the order of *Lactobacillales*, and their associations within LAB families and the BRD-associated *Pasteurellaceae* family in cattle entering the feedlot. The relative abundance of the *Lactobacillales* order increased significantly following the first week of feedlot placement and remained more relatively abundant on day 14 compared to day 0 at the farm. The families within the LAB order—including *Aerococcaceae, Carnobacteriaceae*, and *Streptococcaceae*—were significantly enriched after feedlot placement. The relative abundance of members of the *Lactobacillaceae* family remained stable during the course of study. Overall, positive correlations were observed among the LAB families, suggesting that cooperative relationships exist among different LAB species in the NP microbiota of feedlot cattle. Although these correlations were not statistically significant, negative Spearman's correlation coefficients were observed for all LAB families with *Pasteurellaceae*. This potentially suggests that the presence of LAB in the nasopharynx may have a competitive exclusion effect on the BRD-associated *Pasteurellaceae* family and is supported by the inhibition assays performed. A larger study with more animals would help determine if a true inverse relationship between LAB families and *Pasteurellaceae* exists in the nasopharynx of cattle.

The tested strains within the families of *Lactobacillaceae, Streptococcaceae*, and *Enterococcaceae* displayed *in vitro* antimicrobial activity against pathogen *M. haemolytica*. This finding, along with the observed inverse associations of LAB families with *Pasteurellaceae*, suggests a role for the LAB in protecting against *M. haemolytica* colonization and proliferation in the respiratory tract. Holman et al. (2015a) observed that the relative abundance of the *Lactobacillaceae* family in the nasopharynx of cattle entering the feedlot was significantly greater in animals that remained healthy compared with those that developed BRD. Furthermore, we recently identified several *Lactobacillus* strains isolated from the nasopharynx of healthy feedlot cattle that were able to inhibit the growth of *M. haemolytica* (Amat et al., 2016). The present study suggests that bacteria other than those within the *Lactobacillus* genus are capable of inhibiting *M. haemolytica*, and offers insight into microbial competition within the upper respiratory tract of cattle. It also suggests that bacteria from the bovine respiratory tract may have potential for use as intranasal probiotics or bacterial therapeutics to modulate NP microbiota. Species within the *Lactobacillus* genus have been used as probiotics for both humans and livestock (Fijan, 2014). While certain strains of *Enterococcus* are pathogenic, this genus has also been used in livestock production as a direct fed microbial (EFSA, 2012). However, caution should also be applied when assessing LAB as a single group. For example, members of the *Streptococcus* genus are capable of degrading mucous which may adversely affect host innate immunity (Derrien et al., 2010). In addition, although *Streptococcus* displayed a negative correlation with the *Pasteurellaceae* family on day 0 at the farm, there was a positive association between the two taxa after feedlot placement. Therefore, further research is warranted to investigate *Streptococcus* spp. in the respiratory tract of feedlot cattle.

In conclusion, the composition and structure of the NP microbiota underwent significant changes following commingling at the auction market and up to 12 days after feedlot placement. In particular, the NP microbiota at day 0 and at subsequent sampling times appeared to become more dissimilar. Many of these changes were driven by potential associations among several genera including increases in *Acinetobacter, Bifidobacterium, Corynebacterium, Prevotella*, and *Psychrobacter*, and decreases in *Moraxella, Mycoplasma*, and *Pasteurella*. Despite an increase in microbial richness and the relative abundance of certain genera, similar increases were not observed for BRD-associated pathogens by either sequencing or culturing. This suggests that healthy, low risk calves that have been weaned more than 40 days prior to marketing and feedlot placement, are relatively resilient against these pathogens up to 12 days after feedlot placement. The LAB families were also more relatively abundant in the first week of feedlot placement compared to the farm. Most LAB families were negatively associated with BRD-associated *Pasteurellaceae* family, which includes several BRD pathogens. Some LAB strains were also determined to inhibit the growth of *M. haemolytica in vitro*. Overall, the results of this study suggest that the NP microbiota of calves is dynamic with an increase in microbial richness following transport to an auction market and feedlot. In addition, we provided evidence of potential cooperation and exclusion taking place in the respiratory tract of cattle which may be useful for developing microbial-based strategies to mitigate BRD. Similar analysis of high-risk, immunocompromised cattle is also warranted.

## Data Availability

The datasets generated for this study can be found in the Sequences were submitted to the NCBI sequence read archive under BioProject accession PRJNA296393: BioSamples SAMN04102441–SAMN04102444; SAMN04102453–SAMN04102464; SAMN04102473–SAMN04102492; SAMN04102524–SAMN04102531; SAMN04102540–SAMN04102547.

## Ethics Statement

The calves used in this study were cared for in accordance to the guidelines set by the Olfert et al. (1993) and all experimental procedures involving cattle were approved by the Animal Care Committee of the Lethbridge Research and Development Centre, Agriculture and Agri-Food Canada.

## Author Contributions

TA and ET conceived the study and provided guidance and support for performing the experiment, data interpretation, and manuscript writing. SA designed experiments, collected samples, performed lab work, and analyzed the data. DH performed 16S rRNA gene sequencing analysis and analyzed and interpreted the data. TS assisted with statistical analysis and manuscript preparation. SA and DH wrote the manuscript. All authors read and approved the final manuscript.

### Conflict of Interest Statement

The authors declare that the research was conducted in the absence of any commercial or financial relationships that could be construed as a potential conflict of interest.
