# Supplementary material for: Evaluation of the Nasopharyngeal Microbiota in Beef Cattle Transported to a Feedlot, With a Focus on Lactic Acid-Producing Bacteria
Source: Front Microbiol. 2019 Sep 6;10:1988. doi: 10.3389/fmicb.2019.01988 (PMC6743003; doi:10.3389/fmicb.2019.01988)
Supplement: Supplementary file 2 [file Presentation_1.PPTX]

## Slide 1
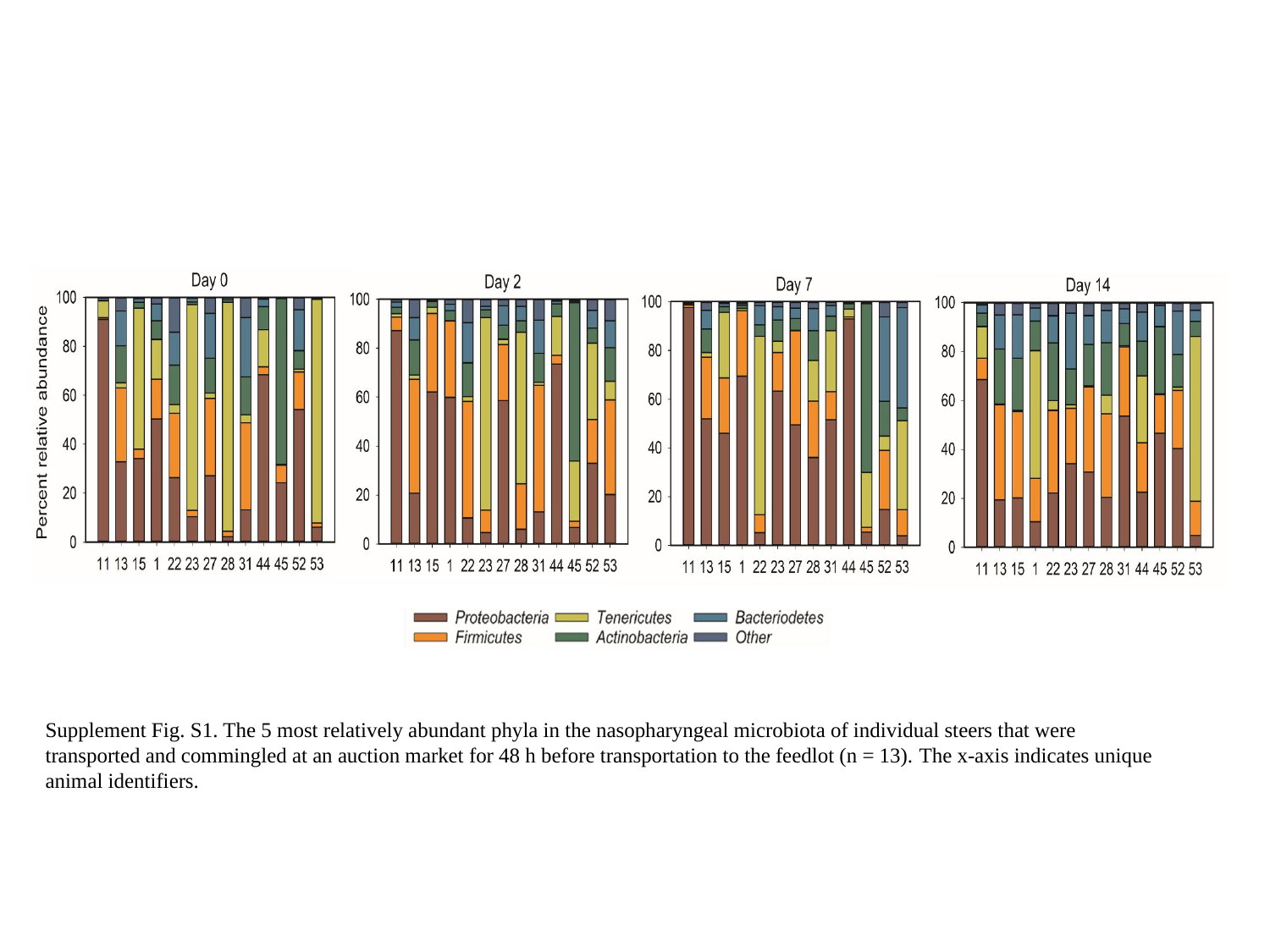

Supplement Fig. S1. The 5 most relatively abundant phyla in the nasopharyngeal microbiota of individual steers that were transported and commingled at an auction market for 48 h before transportation to the feedlot (n = 13). The x-axis indicates unique animal identifiers.
