# Supplementary material for: Evaluation of the Nasopharyngeal Microbiota in Beef Cattle Transported to a Feedlot, With a Focus on Lactic Acid-Producing Bacteria
Source: Front Microbiol. 2019 Sep 6;10:1988. doi: 10.3389/fmicb.2019.01988 (PMC6743003; doi:10.3389/fmicb.2019.01988)
Supplement: Supplementary file 3 [file Presentation_2.PPTX]

## Slide 1
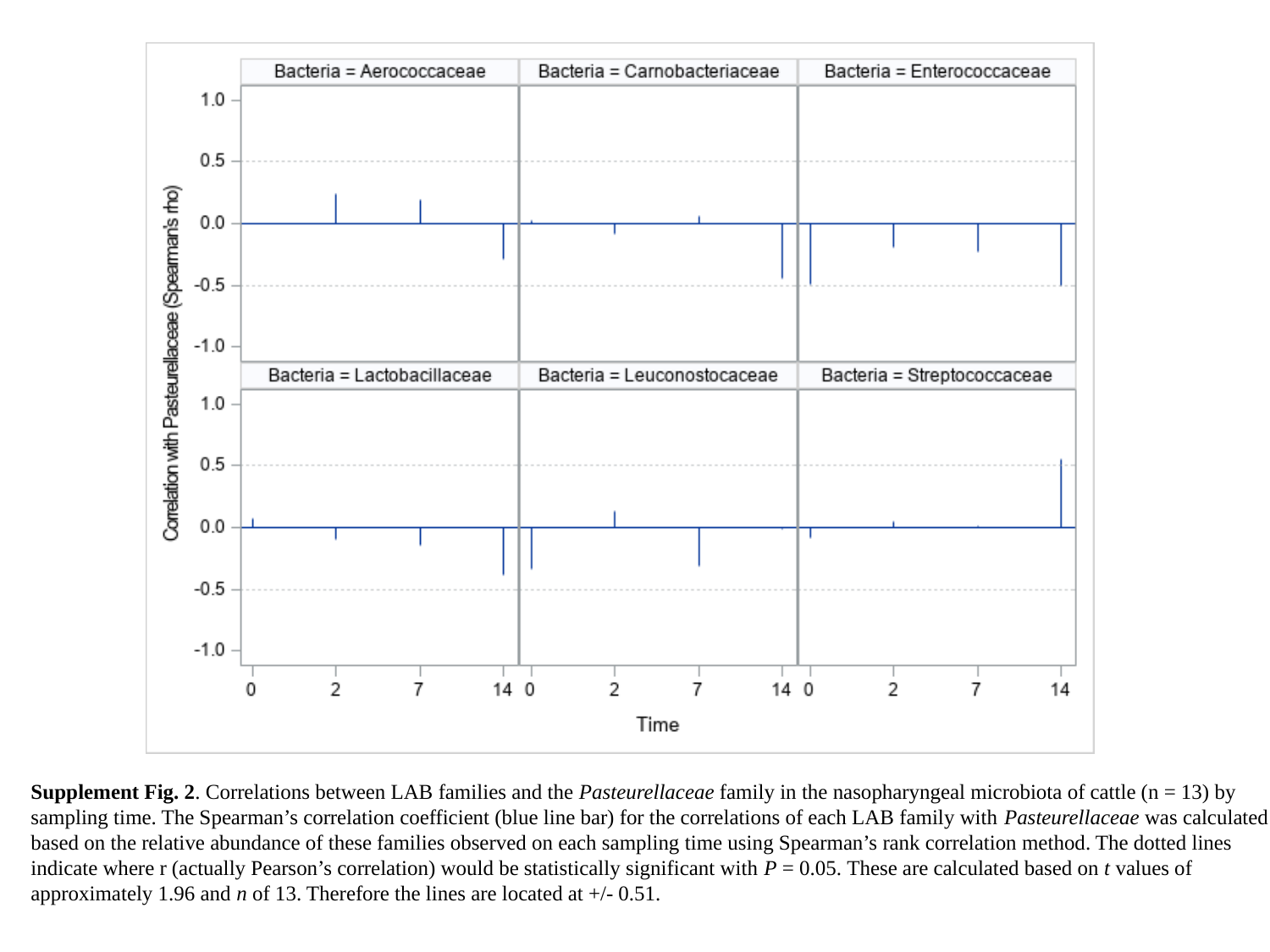

Supplement Fig. 2. Correlations between LAB families and the Pasteurellaceae family in the nasopharyngeal microbiota of cattle (n = 13) by sampling time. The Spearman’s correlation coefficient (blue line bar) for the correlations of each LAB family with Pasteurellaceae was calculated based on the relative abundance of these families observed on each sampling time using Spearman’s rank correlation method. The dotted lines indicate where r (actually Pearson’s correlation) would be statistically significant with P = 0.05. These are calculated based on t values of approximately 1.96 and n of 13. Therefore the lines are located at +/- 0.51.
